# Calibrating the Impressed Anodic Current Density for Accelerated Galvanostatic Testing to Simulate the Long-Term Corrosion Behavior of Buried Pipeline

**DOI:** 10.3390/ma14092100

**Published:** 2021-04-21

**Authors:** Yoon-Sik So, Min-Sung Hong, Jeong-Min Lim, Woo-Cheol Kim, Jung-Gu Kim

**Affiliations:** 1 School of Advanced Materials Science and Engineering, Sungkyunkwan University, Suwon 16419, Korea; soy4718@skku.edu (Y.-S.S.); smith803@skku.edu (M.-S.H.); alsdl0311@skku.edu (J.-M.L.); 2 Technical Efficiency Research Team, Korea District Heating Corporation, 92 Gigok-ro, Yongin 06340, Korea; kwc7777@kdhc.co.kr

**Keywords:** galvanostatic test method, underground infrastructure, long-term corrosion, carbon steel

## Abstract

Various studies have been conducted to better understand the long-term corrosion mechanism for steels in a soil environment. Here, electrochemical acceleration methods present the most efficient way to simulate long-term corrosion. Among the various methods, galvanostatic testing allows for accelerating the surface corrosion reactions through controlling the impressed anodic current density. However, a large deviation from the equilibrium state can induce different corrosion mechanisms to those in actual service. Therefore, applying a suitable anodic current density is important for shortening the test times and maintaining the stable dissolution of steel. In this paper, to calibrate the anodic current density, galvanostatic tests were performed at four different levels of anodic current density and time to accelerate a one-year corrosion reaction of pipeline steel. To validate the appropriate anodic current density, analysis of the potential vs. time curves, thermodynamic analysis, and analysis of the specimen’s cross-sections and products were conducted using a validation algorithm. The results indicated that 0.96 mA/cm^2^ was the optimal impressed anodic current density in terms of a suitable polarized potential, uniform corrosion, and a valid corrosion product among the evaluated conditions.

## 1. Introduction

With the recent development of general industry, the demand for various types of pipeline has increased. Numerous infrastructures have been built in downtown underground areas. Most of these underground infrastructures are aimed at achieving long-term use, since they are generally difficult to maintain or replace. Therefore, it is essential to verify the long-term corrosion behavior of buried metallic structures.

In fact, despite the various developments, underground structural failures continue to occur [1,2,3]. Here, the corrosion of metallic infrastructures in underground soil is a major issue that presents numerous safety and economic concerns [4,5,6,7,8]. In short, pipelines can be damaged by corrosion, which can lead to the failure of the structures within a soil environment. However, it is difficult to detect the failure of a large underground system, which means understanding the long-term corrosion behavior is crucial to mitigating unpredictable failures. As such, various studies have been conducted on the long-term corrosion mechanism for metals in a soil environment. The majority of these studies involved the use of immersion tests to analyze the corrosive characteristics of the metals [9,10,11,12]. However, obtaining the results of this type of test requires a long period of time (at least several months). It is also difficult to maintain the same environmental conditions during the entire test period, making it difficult to yield reproducible results. Since an immersion test is not suitable for evaluating the long-term corrosion properties of materials, an appropriate acceleration test must be considered. Here, electrochemical acceleration methods present the most effective approach for simulating long-term corrosion. Among the various methods, galvanostatic tests allow for accelerating the surface chemical reactions through controlling the impressed anodic current density. It is thus a suitable method for long-term corrosion studies.

The impressed anodic current density and the time can be calculated using Faraday’s law [13]. Applying an appropriate anodic current density is the key factor here, since the expected corrosion reaction cannot be achieved if the impressed density is too high [14]. Meanwhile, accelerated corrosion testing methods must allow for shortening the test time and inducing the same mechanism of degradation as that in actual service. However, there exists no international standard for these tests. With this in mind, this study was aimed at providing an academic standard for anodic current density that can be applied to accelerate and simulate the long-term corrosion of metals in a soil environment. Thus, a potentiodynamic (PD) polarization test was conducted to determine the corrosion current density for carbon steel in synthetic groundwater. To determine the most appropriate anodic current density, galvanostatic (GS) tests were performed at four different anodic current densities using an acceleration period that represented the one-year corrosion reaction of a specific pipeline. Each anodic current density value was determined according to the corrosion current density, which was obtained from the PD polarization measurements and through the use of Faraday’s law. Meanwhile, an optical microscope (OM) was used to observe the surface and cross-section morphologies of the specimens, while X-ray diffraction (XRD) analysis was used to confirm the species of each oxide following the galvanostatic experiments.

## 2. Materials and Methods

### 2.1. Specimen and Solution

The specimen was cut into cuboid-shaped pieces with dimensions of 10 × 10 × 5 mm^3^. The chemical composition of the tested pipeline steel is presented in Table 1 (ASTM A 139), while the composition of the synthetic soil solution is presented in Table 2. The results were obtained from three soil environment sites close to an operating pipeline.

### 2.2. Electrochemical Analysis to Optimize the Impressed Anodic Current Density for GS Testing

To evaluate the corrosion resistance of pipeline steel, a PD polarization test was conducted using a multi-potentiostat/galvanostat instrument (VMP-2, Bio-Logic Science Instruments, Seyssinet-Pariset, France). Meanwhile, a three-electrode cell was constructed using pipeline steel as the working electrode (WE), two pure graphite rods as the counter electrode (CE, Qrins, Seoul, Korea), and a saturated calomel electrode as the reference electrode (RE, Qrins). Prior to conducting the electrochemical tests, the specimens were abraded with 600-grit silicon carbide paper. These prepared steel surfaces were then covered with silicone rubber, leaving an area of 0.25 cm^2^ unmasked before they were exposed to a synthetic soil solution at 60 °C under aerated conditions, and then rinsed with ethanol and finally dried using nitrogen gas. Prior to all the electrochemical tests, the specimens were immersed in a test solution for 3 h to attain a stable surface. The PD polarization measurements were performed at a potential sweep of 0.166 mV/s from an initial potential of −250 mV vs. an open circuit potential (OCP) up to a final potential of 0 V_SCE_. A GS test was then performed to accelerate corrosion reaction of steel after 3 h of OCP measurements at four different anodic current densities. Each of the impressed anodic current density values was determined according to the corrosion current density, which was obtained from the PD polarization measurements.

### 2.3. Surface Analysis

An OM (SZ61TRC, Olympus Korea Co., Seocho-gu, Seoul, Korea) was used to observe the surface morphology and the cross-section of each specimen, while each oxide product was analyzed via XRD (Rigaku Ultima III X-ray diffractometer, Tokyo, Japan) analysis with Cu Kα_1_ radiation (λ = 1.54056 Å) over a 2θ range of 20°–70°, using a step-size of 0.017° and a step-time of 1 s, to confirm the species of each oxide following the galvanostatic experiments.

## 3. Results and Discussion

### 3.1. Corrosion Behavior of Pipeline Steel in a Synthetic Soil Solution

**Figure 1:**
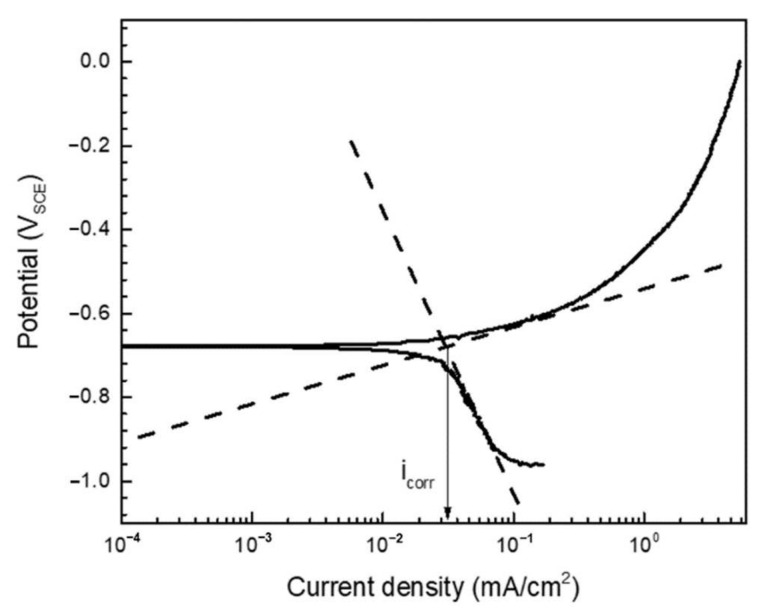
The PD polarization curve of steel in a synthetic soil solution at pH 6.4 and a temperature of 60 °C.

The PD polarization curve related to a synthetic soil solution at a pH of 6.4 and a temperature of 60 °C is shown in Figure 1, while the PD results are summarized in Table 3.

To calculate the corrosion current density, the Tafel extrapolation method (as described in the equation below) was applied. Here, Equation (1) describes the linear relationship between the over-potential and the log scale current density [14,15]:

ƞ = a *±* β_a,c_log|i| (1)

where a = −β_a_log(i_0_) or β_c_log(i_0_), β_a_ ≅ (RT/(1 − α)nF) is the Tafel slope of the anodic polarization curve, β_c_ ≅ (RT/αnF) is the Tafel slope of the cathodic polarization curve, i_0_ is the exchange current density, α is the charge transfer coefficient, n is the charge number, R is the gas constant (8.314 J/[mol∙K]), and T is the absolute temperature [K]. From Equation (1), a linear relationship was derived, and the corrosion current density was measured as approximately 27.96 µA/cm^2^ according to the Tafel extrapolation in the PD polarization curve (Figure 1). No passivation behavior of the anodic polarization curve was observed, which means that in a synthetic soil solution, pipeline steel will be homogeneously corroded.

### 3.2. Corrosion Acceleration Using the GS Method

The mass loss of pipeline steel can be calculated for each current and experiment time by applying Faraday’s law. The mass loss by PD test for the one-year corrosion of pipeline steel is given in the following Equation (2):

m = ita/nF = (27.96 μA/cm^2^ × 1 year × 55.84)/(2 × 96500 C) = 0.255 g/cm^2^ (2)

where m is mass loss, i is corrosion current density, F is Faraday’s constant (96,500 C/equivalent), n is the number of equivalents exchanged, a is the atomic weight, and t is time [16]. To accelerate the one year of corrosion, the impressed anodic current density and test time were set accordingly. The impressed anodic current densities were selected to investigate a wide range as possible, starting from the maximum output (24 mA, 3435.29 times faster than corrosion rate) of the potentiostat instrument (VMP-2) before being reduced to 0.024 mA (3.43 times faster than corrosion rate) in 1/10 stages. Meanwhile, the exposure times were also determined to maintain the same theoretical metal loss for each test. With the reduction in exposure time, the acceleration coefficient, which is the ratio between impressed anodic current density and corrosion current density, increased sharply. All of these variables are detailed in Table 4.

**Figure 2:**
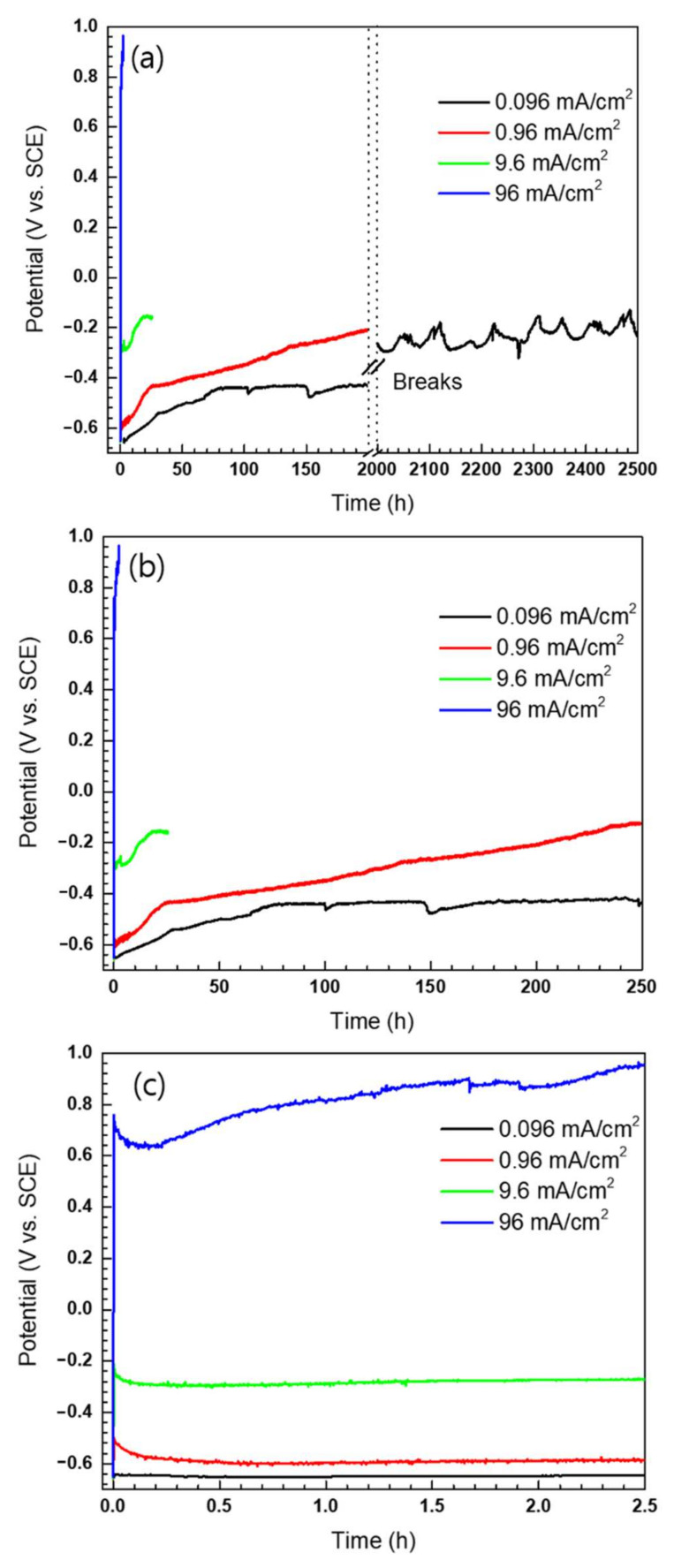
Potential (V_SCE_) vs. time (h) curves during the GS test at 60 °C. The shape of the graph follows the Sand equation. (**a**) Entire curves with breaks; (**b**) graph from 0 to 250 h; (**c**) graph from 0 to 2.5 h.

The logarithmic shape of the curves in Figure 2 follows Equation (3), known as the Sand equation [17]. This equation defines the quantitative relationship between the impressed anodic current density and time:

(iτ^1/2^)/(C_0_*) = (nFD_0_^1/2^π^1/2^)/2 (3)

where i is the impressed current density, τ is the transition time, C_0_* is the bulk concentration of the reactant, n is the coefficient number, and D_0_ is the diffusion coefficient of the reactant. When the impressed current can no longer be supported by the intended metal dissolution reaction, the potential changes to an alternative electron-transfer reaction [17]. However, in most of the accelerated GS corrosion tests, the reactants were the metal itself. Therefore, the probability that the concentration of the reactants reaches zero is extremely low. In Figure 2, since the transition point could not be observed, i.e., the potential vs. time curve exhibited a logarithmic curve shape, the GS tests were appropriately performed under all conditions. Nevertheless, there were differences in the stability of the curve shape for each condition. As shown in Figure 2a, the wavering potential curve was recorded only at the slowest reaction rate of 0.096 mA/cm^2^. When the reaction rate is slower, oxides will have more opportunity to adsorb onto the electrode surface. Hence, it can be expected that there will be an obstruction of the reaction area comprising the laminated oxide layer. Consequently, in this experiment, the 0.096 mA/cm^2^ condition appeared to be invalid for accelerating a homogeneous corrosion using the GS method.

**Figure 3:**
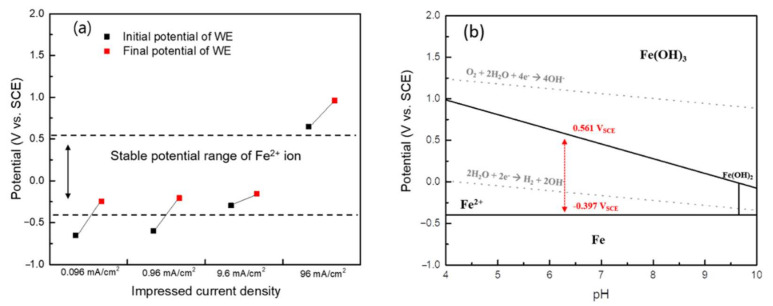
(**a**) Initial and final potentials during the GS test, and (**b**) Pourbaix diagram of an Fe-H_2_O system (activity of Fe^2+^: 10^−6^, 60 °C).

Meanwhile, the measured potential of each condition was different depending on the impressed anodic current density. As the impressed anodic current density increased, the WE potential increased to more positive values and was generated at a higher current during a relatively short period of time, with the initial and final values of the measured potentials shown in Figure 3a. Here, it was clear that the measured WE potential depended on the impressed anodic current density. Therefore, thermodynamic analysis was then conducted to verify the electrochemically accelerated reaction. The reactions and the Nernst equations that primarily occur in the Fe-H_2_O system at 60 °C are described in terms of Equations (4)–(6) [13], while these are also presented in terms of a pH-potential diagram in Figure 3b.

Fe^2+^ + e^−^ = Fe, ɛ_[Fe2+/Fe]_ = −0.199 + 0.033 log[Fe^2+^], [V_SCE_] (4)

Fe(OH)_3_ + 3H^+^ + e^−^ = Fe^2+^ + 3H_2_O, pH = 6.65 − 0.5 log[Fe^2+^] (5)

Fe(OH)_2_ + 2H^+^ = Fe^2+^ + 2H_2_O, ε_[Fe2+/Fe(OH)3)]_ = 1.298 − 0.177 pH − 0.066 log[Fe^2+^], [V_SCE_] (6)

In Figure 3b, the metal ions appeared to be stable in the range of –0.397 to 0.561 V_SCE_ when the soluble ion activity was 10^−6^ at pH = 6.4 and 60 °C, as shown in the Pourbaix diagram. Meanwhile, as Figure 3a shows, the 0.096 and 0.96 mA/cm^2^ current densities had initial and final potentials of −0.651 and −0.244 V_SCE_, −0.597 and −0.206 V_SCE_, respectively, while the 9.6 mA/cm^2^ current density had an initial potential of −0.295 and a final potential of −0.153 V_SCE_. These three conditions indicated the region where the metal ions were both stable and sensitive to corrosion (see Figure 3b). As such, the corrosion of carbon steel will be accelerated accordingly. However, the 96 mA/cm^2^ current density had extreme potentials, with an initial potential of 0.648 V_SCE_ and a final potential of 0.960 V_SCE_. In this potential range, a metal-dissolution reaction is no longer stable, meaning the thermodynamic stability of the pipeline steel will change from iron to iron oxide/hydroxide ions, based on the Pourbaix diagram [18]. In addition, an oxygen-evolution reaction may occur close to 1 V_SCE_. As such, the 96 mA/cm^2^ condition, as a current value for accelerating the corrosion process, cannot be reasonably accepted.

Meanwhile, Table 5 shows the parameters following the accelerated tests for corrosion over a one-year period. During the GS test, the electrode was exposed to a water-based solution, with the primary reaction being the production of electrons via metal dissolution. However, a self-corrosion reaction also occurred [19], which consumed the electrons generated by the dissolution of iron from the WE surface [20]. Due to this self-corrosion reaction, the generated electrons were not completely transported to the CE; rather, they reacted with the water and oxygen at the WE’s surface. As a result, the mass loss in the GS acceleration test became larger than the theoretical mass loss calculated using Faraday’s law. As such, in electrochemically accelerated tests, the self-corrosion ratio in relation to the corrosion rate could be around 20–30%, as shown in Table 5. Nevertheless, the electrochemical acceleration test had the advantage of a significantly reduced testing time. For example, while with the 0.96 mA/cm^2^ condition, a 28.9% self-corrosion ratio was indicated, the testing time was generally reduced from one year to around 10 days. Thus, the electrochemical acceleration method still has merits despite the self-corrosion aspect.

### 3.3. Analysis of Surfaces

**Figure 4:**
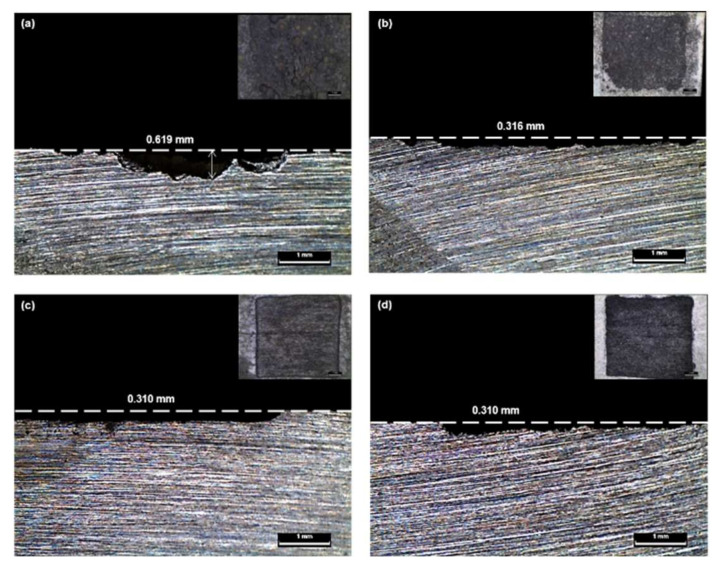
Cross-sectional images following the galvanostatic test at different applied current densities: (**a**) 0.096 mA/cm^2^; (**b**) 0.96 mA/cm^2^; (**c**) 9.6 mA/cm^2^; (**d**) 96 mA/cm^2^.

Cross-sectional images of the tested specimens obtained using the OM are shown in Figure 4. Most of the images clearly indicated a uniform corrosion. However, at the lowest anodic current density of 0.096 mA/cm^2^, different behavior, which indicated localized corrosion, was observed. As shown in Figure 2, an unstable potential was recorded at the anodic current density of 0.096 mA/cm^2^. As noted above, when the reaction rate is slower, oxides will have more opportunity to adsorb onto the electrode’s surface. Hence, most of the iron oxides produced via the self-corrosion reaction covered the reaction area at the anodic current density of 0.096 mA/cm^2^. Consequently, an accelerated corrosion reaction occurred at the localized site of the narrow reaction area, due to the layer of iron oxide formed on the surface.

**Figure 5:**
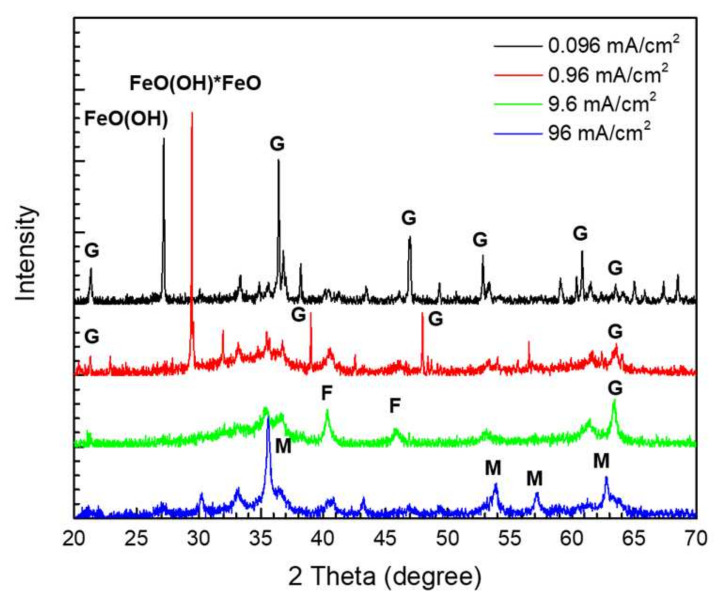
The XRD results following the galvanostatic tests based on current density: G: goethite (α-FeO[OH]); F: ferrihydrite (Fe_2_O_3_∙0.5H_2_O); M: magnetite (Fe_3_O_4_).

Meanwhile, phase analysis of the oxides under each anodic current density condition was conducted using the XRD instrument, with the major phase of the oxides shown in Figure 5. Here, the corrosion product of the lower anodic current density group was mainly comprised of goethite. However, the higher anodic current density condition (96 mA/cm^2^) showed different XRD peaks, which represented ferrihydrite and magnetite. According to the existing literature, the main iron oxide phase in general soil environments is goethite [10,20,21,22,23,24]. Furthermore, the XRD results at the 0.096 and 0.96 mA/cm^2^ conditions were similar to those obtained for actual environment iron oxides in previous studies. Therefore, these conditions accurately reflected the long-term corrosion behavior of buried pipeline.

### 3.4. Validation of the Galvanostatically Accelerated Testing

**Figure 6:**
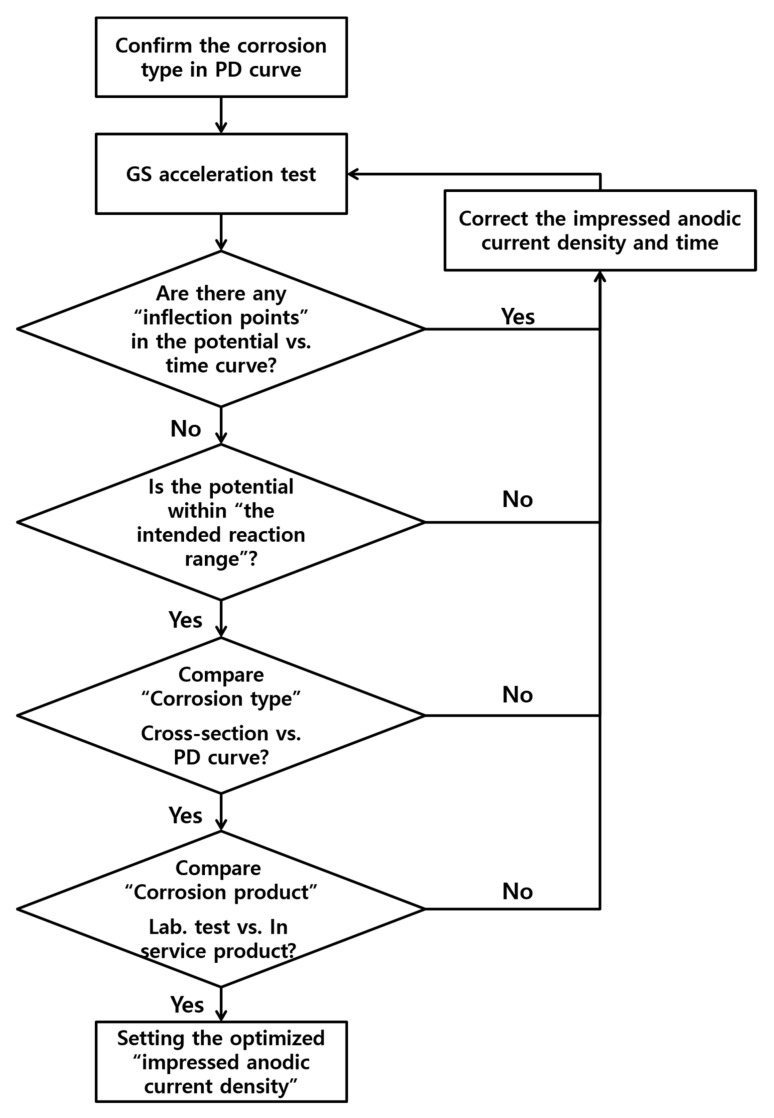
Flowchart showing the validation process for the GS accelerated testing.

To establish an appropriate impressed anodic current density for the accelerated test, a validation process was conducted for all tests, based on the process shown in Figure 6. This process was initially implemented to assess the inflection points in the potential vs. time curves observation, and thus to confirm the effect of the environment during the test. In the galvanostatic test, all the curves exhibited a stable logarithmic shape, indicating that the pipeline steel reacted well. Meanwhile, the validation process involved comparing the measured potentials with the Pourbaix diagram. Here, the majority of the conditions are within a thermodynamically stable Fe ion range. However, the 96 mA/cm^2^ condition departed from this range and was thus excluded from the valid current density range. Next, the validation process was continued in terms of the cross-sectional images of the steel pipeline specimen to confirm the presence of uniform corrosion, which was indeed clearly confirmed by the majority of the images. However, the lowest anodic current density (0.096 mA/cm^2^) indicated different corrosion behavior (localized corrosion) and was thus also excluded from our determination of a valid anodic current density. Finally, the validation process involved analyzing the corrosion product using XRD analysis. Here, 0.96 mA/cm^2^ was found to be the optimal anodic current density for reproducing long-term corrosion behavior.

## 4. Conclusions

In this study, the impressed anodic current density for a GS test aimed at determining long-term corrosion behavior was evaluated using an electrochemical test, OM observation, and XRD analyses. To verify the accelerated test, analysis of the potential vs. time curves, thermodynamic analysis, and analysis of the cross-sections and products of the specimen were performed. During the GS test, based on the laminated oxide layer, the most unstable potential form was recorded at the slowest reaction rate (0.096 mA/cm^2^). Meanwhile, the highest anodic current density (96 mA/cm^2^) demonstrated extreme potentials that were out of the Fe ion’s stable range. The majority of the OM images clearly indicated uniform corrosion. However, the slowest condition at the anodic current density of 0.096 mA/cm^2^ indicated localized corrosion. The XRD peaks at the 0.096 and 0.96 mA/cm^2^ conditions corresponded to a corroded buried pipeline product (goethite), while the 9.6 and 96 mA/cm^2^ conditions indicated the presence of different oxides. In conclusion, the anodic current density of 0.96 mA/cm^2^ was found to be the most suitable for conducting the GS acceleration testing of carbon steel in a soil environment. Based on this finding, an appropriate validation process was established for an accelerated corrosion test aimed at predicting long-term corrosion lifetimes. Furthermore, it is expected to help determine the reliable impressed anodic current density by applying the validation process to design accelerating metal corrosion.

## Figures and Tables

**Table 1:** Composition of the tested steel specimen.

C	Mn	P	S	Fe
0.25	1.00	0.04	0.04	Balance

**Table 2:** Composition of the tested solution.

pH	Temperature	CaCl_2_ (ppm)	MgSO_4_·7H_2_O (ppm)	NaHCO_3_ (ppm)	H_2_SO_4_ (ppm)	HNO_3_ (ppm)
6.4	60 °C	133.2	59	208	85	22.2

**Table 3:** Results of the PD (potentiodynamic) polarization measurements.

E_corr_ (mV_SCE_)	i_corr_ (µA/cm^2^)	β_a_ (mV/decade)	β_c_ (mV/decade)
−678.03	27.96	113.6	579.6

**Table 4:** Experimental conditions and calculated variables.

Impressed Anodic Current Density (mA/cm^2^)	Exposure Time (h)	Mass Loss by PD Test (g/cm^2^)	Anodic Dissolution Rate (mm/y)	Acceleration Coefficient
0.096	2551.35	0.255	1.14	3.43
0.96	255.14		11.44	34.34
9.6	25.51		114.39	343.35
96	2.55		1143.90	3435.29

**Table 5:** Measured parameters of the one-year corrosion-accelerated specimen as a function of impressed anodic current density.

Impressed Anodic Current Density (mA/cm^2^)	Mass Loss by GS Test (g/cm^2^)	Corrosion Rate ^1^ (mm/y)	Self-Corrosion Rate ^2^ (mm/y)	Self-Corrosion Ratio ^3^ (%)
0.096	0.364	1.63	0.49	30.06
0.96	0.360	16.09	4.65	28.90
9.6	0.336	150.23	35.85	23.86
96	0.332	1485.05	341.15	22.97

^1^ Corrosion rate: calculated values from mass loss by GS test. ^2^ Self-corrosion rate: difference between corrosion rate by PD test and GS test. ^3^ Self-corrosion ratio: (self-corrosion rate/corrosion rate) × 100.

## Data Availability

Data is contained within the article material.
